# Feeding back the results of trials to the families of participants who have died: methodological considerations from the bracelet study (bereavement and randomised controlled trials)

**DOI:** 10.1186/1745-6215-14-S1-O32

**Published:** 2013-11-29

**Authors:** Claire Snowdon, Peter Brocklehurst, Robert Tasker, Martin Ward Platt, Diana Elbourne

**Affiliations:** 1 Medical Statistics Department, London School of Hygiene and Tropical Medicine, London, UK; 2 National Perinatal Epidemiology Unit, University of Oxford, Oxford, UK; 3 Institute for Women’s Health, University College London, London, UK; 4 Department of Neurology, and Anaesthesia (Pediatrics), Harvard Medical School, Boston, USA; 5 Newcastle Neonatal Service, Royal Victoria Infirmary, Newcastle-upon-Tyne, UK

## 

Increasingly trial results are fed back to participants and their families, but there is little research about this process if the participant dies. This issue was explored in the BRACELET Study (Bereavement and Randomised Controlled Trials) (http://www.bracelet-study.org.uk). We conducted 30 interviews with 51 bereaved parents of babies who were entered into one of five neonatal intensive care trials, and 58 clinicians and/or trial team members.

Bereaved parents expressed a range of views but almost unanimously supported offering parents trial results. Results were important for information, but also as an acknowledgement of loss and contributions to research, as connections to their baby and for commemoration and consolation. Parents thought that feedback would help them feel that their baby was not forgotten but valued as an individual, conferring meaning to a short life.

Feedback is however not straightforward. Trials may take a long time to recruit, analyse and report results. Parental circumstances can change over time: those disinclined to consider receiving results at an earlier stage of bereavement may change their views; those initially interested may become less so. The capacity of a trial team to respond may also change: dedicated trial staff may no longer be in post, funds for feedback may be limited and parental addresses may no longer be current.

Further careful studies of feedback of results are needed in a variety of trial settings to understand how trial teams manage this situation, and to explore how results are received and understood by both bereaved and non-bereaved families.

